# Human Papillomavirus and Cervical Cancer Knowledge, Perceptions, and Screening Behavior: A Cross-Sectional Community-Based Survey in Rural Philippines

**DOI:** 10.31557/APJCP.2020.21.11.3145

**Published:** 2020-11

**Authors:** Atsuko Imoto, Sumihisa Honda, Erlidia F Llamas-Clark

**Affiliations:** 1 *School of Tropical Medicine and Global Health, Nagasaki University, Nagasaki, Japan.*; 2 *Department of Community-based Rehabilitation Sciences, Graduate School of Biomedical Sciences, Nagasaki University, Nagasaki, Japan. *; 3 *Section of Ultrasound, Department of Obstetrics and Gynecology, University of Philippines Manila*,* Philippine General Hospital, Philippines. *

**Keywords:** Cervical cancer screening, knowledge, perception, the Philippines

## Abstract

**Background:**

Cervical cancer is the second most common cancer among women in the Philippines. Cervical cancer screening is an effective method to reduce incidence. However, screening utilization is limited. This study aims to assess human papillomavirus (HPV) and cervical cancer knowledge, perceptions, and screening utilization, and to investigate factors influencing screening utilization among rural women in the Philippines.

**Methods:**

This cross-sectional community-based study was conducted among 338 rural women aged 20–50 years, with a child under 5 years old registered in one of four public rural health centers in Tacao Island, Masbate Province in October 2017. A questionnaire administered via face-to-face interviews elicited information about demographic characteristics, knowledge, perceived susceptibility and perceived severity of HPV and cervical cancer, and cervical cancer screening utilization.

**Results:**

Mean age of participants was 32.5 years. Only 13.9% of participants had ever had cervical cancer screening. Although most women had heard of cervical cancer screening, their knowledge about the cause, risk factors, and preventive measures of HPV and cervical cancer was limited. Older age and higher education status were significantly associated with screening utilization. However, knowledge and perceived susceptibility and severity showed no association. The main reason for having screening was due to a health professional’s request or recommendation, and the reasons for not having screening were cost, not having symptoms, and fear of pain or discomfort and/or embarrassment during the procedure.

**Conclusions:**

Health education must increase knowledge about HPV and cervical cancer and screening among women, including the nature and progression of cervical cancer, benefits of screening, screening cost, and screening procedure. Health care providers have an important role in educating and motivating women to undergo screening.

## Introduction

Cervical cancer is the second most common cancer among women in developing countries. Human papillomavirus (HPV) is the causative agent of cervical cancer, which is transmitted through sexual contact (Bosch et al., 2002). In the Philippines, cervical cancer is the second most common type of cancer among women, causing 6,670 cases and 2,832 deaths every year (Bruni L, 2017). The age-standardized incidence rate is 16.0 per 100,000 in Philippines, which is similar to the 16.3 per 100,000 in Southeast Asia (Bruni L, 2017). Cervical cancer is usually diagnosed at advanced stages. Among 1,580 Filipino patients randomly selected from national cancer registries in the National Capital Region from 1993 to 2002, 23.9% of them were diagnosed at stage I, 33.1% at stage II, and 43.1% at stage III or above (Redaniel et al., 2009).

Cervical cancer is preventable through universal access to comprehensive screening and treatment programs. Screening and early detection reduce the incidence and mortality of cervical cancer. Pap smear is widely used as a screening tool for preventing cervical cancer. However, in limited resource settings, it is not feasible due to lack of trained personnel and laboratory facilities. Therefore, alternative cost-effective methods are offered, such as visual inspection with acetic acid (VIA) (Sherris et al., 2009). In developed countries, organized screening programs have been successful in reducing the incidence and mortality of cervical cancer. However, in many developing countries, like Philippines, cervical cancer screening remains to be opportunistic.

Pap smear was widely introduced in the Philippines in 1997 (DOH Philippines, 1997) and cervical cancer prevention advocacy program was initiated in 2003 to raise awareness about cervical cancer prevention during the month of May every year (Office of the President, 2003). In 2005, the policy was shifted to a single-visit approach using VIA followed by cryotherapy, considering the low utilization of Pap smear (DOH Philippines, 2005; Guerrero et al., 2015). With the new methods to prevent cervical cancer, it is important to understand the level of knowledge and perceptions of cervical cancer that may contribute to screening behavior, and factors associated with screening utilization among women, for effective cancer prevention program. It is assumed that women from rural regions tend to have disadvantaged access to health care services due to inadequate health care infrastructure. Thus, this study aimed to assess the knowledge and perceptions about HPV and cervical cancer and cervical cancer screening utilization, and to investigate the factors influencing screening utilization, among rural women in the Philippines.

## Materials and Methods

A cross-sectional survey was conducted in October 2017 at four public rural health centers located in four municipalities (Batuan, San Fernando, Monreal, San Jacinto) of Ticao Island, Masbate Province, Bicol region. The Bicol region consists of 6 provinces with a total population of about 5.8 million in 2015 (PSA, 2015). In the region, cancer is the third cause of mortality following by disease of heart and pneumonia (DOH Philippines-Center for Health Development-Bicol Legazpi City, 2013) and life expectancy at birth for females is 73.8 years (PSA, 2000). Ticao is one of three main islands of Masbate Province with a total population of about 95,000 in 2015 (PSA, 2015). The Department of Health provides basic primary care services through rural health centers and barangay (i.e. village) health stations located in municipalities and specialized ambulatory care/in-patient care services through a district hospital on Ticao Island.

A total of 600 participants, 150 from each rural health center, were selected randomly from a list of mothers of children under 5 years old registered at each health center. Mothers aged 20–50 years were included; those who had undergone a total hysterectomy during the survey or before, or had been diagnosed with cervical cancer were excluded. Community health workers who were familiar with residents at the study sites approached mothers and invited them to join the study after they had been given a full explanation of the study objectives. Trained health staff and research assistants obtained informed consent and conducted face-to-face interviews at the health centers.

The questionnaire included participants’ age, education, religion, marital status, employment status, household monthly income, parity, time needed to reach the nearest facility offering cervical cancer screening, and past contraceptive use. Women were also asked about their knowledge, perceived susceptibility, and perceived severity of HPV and cervical cancer using a standardized questionnaire adapted from a previous study (Ingledue et al., 2004). The survey was prepared based on the Health Belief Model (HBM), which has been widely used to understand preventative health behaviors such as cancer screening (Glanz, 2008). The knowledge part consists of 15 multiple-choice questions such as cause, risk factors, and preventive measures. Each question have one correct answer and a score of 1 is given for each correct response. Total scores range from 0 to 15, with higher scores indicating greater knowledge. The perceived susceptibility (e.g., beliefs about the risk of getting HPV or cervical cancer) and perceived severity (e.g., beliefs about the severity of the consequences of HPV or cervical cancer) portions consisted of 15 questions: 6 for perceived susceptibility and 9 for perceived severity. Each question was scored using a 5-point Likert scale ranging from strongly disagree (1) to strongly agree (5), with higher scores reflecting greater perceived susceptibility and severity. Total possible scores ranged from 6–30 and 9–45, respectively. Further, participants were questioned about awareness and sources of information on cervical cancer screening, previous screening, reasons for having or not having screening, and having a family member or friend who has been diagnosed with cervical cancer. The questionnaire was prepared in English, then translated into Filipino. Cronbach’s alpha was 0.65 for knowledge, 0.80 for susceptibility, and 0.67 for severity. 

Descriptive and univariate analyses were conducted using appropriate test statistics and variables associated with the screening behavior with a p-value <0.25 were added to the model. Stepwise logistic regression was performed to identify factors affecting screening behavior. All data was analyzed using Stata/IC version 14.0. Ethics approval were granted by the University of the Philippines, Manila and Nagasaki University, Japan. 

## Results

A total of 338 women aged 20 to 50 years participated in the study. The major reason for non- participation was that they were busy with childcare and housework. Mean age of the participants was 32.5 years with standard deviation (SD) of 7.2. Most women had no formal education or primary education, were Roman Catholic, married or living together with a partner, unemployed, and had more than two children. Nearly all (93.8%) women had ever heard of Pap smear or VIA, and the source of information about screening was mostly from the health facility (71.3%) (Table1a and 1b).

Table 2 shows that the main reasons for undergoing screening were a request or recommendation from a health professional (83.1%) and some concerns about symptoms (10.6%). The main reasons for not having screening were: lack of money (23.0%), having no signs or symptoms (22.7%), fear of pain or discomfort and/or embarrassment during the procedure (16.8%), never heard of or did not understand the meaning of cervical cancer screening (13.1%), and that it had not been promoted promoted (12.7%).

Table 3 shows the differences in characteristics between participants who had or never had screening. Only 47 (13.9%) participants had ever undergone screening. Participants who were aged 30–39 years (p<0.001), had a high school education (p<0.001), were Roman Catholic (p=0.046), and employed (p=0.012) were significantly associated with screening utilization (p<0.05). The mean score of knowledge among participants was 6.4 (SD=2.5). The mean scores of susceptibility and severity among participants were 21.9 (SD=5.1) and 34.2 (SD=5.2), respectively. There were no significant differences in the median scores of knowledge, susceptibility, and severity between the two groups.

From the univariate analysis, age, education, religion, marital status, employment status, contraceptive use, household monthly income, and having a friend or family member diagnosed with cervical cancer were found to have a p-value <0.25. In addition to these variables, knowledge, susceptibility and severity were included in multivariable stepwise logistic regression analysis. The results showed that age 30–39 years (odds ratio (OR)=3.76, 95% confidence interval (CI): 1.53–9.22), age 40 years or more (OR=6.58, 95% CI: 2.47–17.60), and having secondary level education and higher (OR=4.20, 95% CI: 2.10–8.44) were significantly associated with screening utilization (Table 4). 

**Table 1a:** Awareness of Pap Smear or Visual Inspection with Acetic Acid (VIA)

		N	%
Ever heard of Pap smear or VIA	Yes	317	93.8
(n=338)	No	21	6.2

VIA, visual inspection with acetic acid

**Table 1b:** Source of Information about Pap Smear or VIA

		N	%
Source of information about Pap Smear or VIA (n=317)	Health facility	226	71.3
	Friend or family member	73	23
	TV	10	3.2
	Learning institution or other learning opportunities	6	1.9
	Radio	2	0.6

**Table 2:** Participants’ Reasons for Having and not Having Cervical Cancer Screening

		N	%
Reason for having screening (n=47)	Requested or recommended by health professional	39	83.1
	Existence of concerning symptoms vaginal/abdomen	5	10.6
	Requested by health worker	1	2.1
	To check the condition of the cervix	1	2.1
	Missing	1	2.1
Reason for not having screening (n=291)	Lack of money	67	23.0
	No signs or symptoms	66	22.7
	Fear of pain or discomfort and /or embarrassment during the procedure	49	16.8
	Never heard of or do not understand meaning of cervical cancer screening	38	13.1
	Not promoted	37	12.7
	Do not know where service is available	21	7.2
	Scared of result	5	1.7
	Thought unnecessary because no risk of cervical cancer	3	1.0
	Too far to health facility offering testing	2	0.7
	Unclear response	3	1.0

**Table 3:** Comparison between Participants who Ever had Cervical Cancer Screening and Those who Never had Cervical Cancer Screening (N=338)

Characteristics	Total	Ever had cervical cancer screening, N (%)		*P*-value
		Yes (n=47)	No (n=291)	
Age, years				
Mean (standard deviation )	338	37.2 (6.9)	31.7 (7.0)	
20–29	134	7 (14.9)	127 (43.6)	<0.001†
30–39	148	24 (51.1)	124 (42.6)	
≥40	56	16 (34.0)	40 (13.8)	
Education				<0.001‡
No formal education or primary	276	27 (57.4)	249 (85.6)	
Secondary or higher	62	20 (42.6)	42 (14.4)	
Religion				0.046‡
Roman Catholic	312	40 (85.1)	272 (93.5)	
Non-Catholic	26	7 (14.9)	19 (6.5)	
Marital status				0.050‡
Married	222	37 (78.7)	185 (63.6)	
Living with partner	101	7 (14.9)	94 (32.3)	
Never married / widowed	15	3 (6.4)	12 (4.1)	
Employment status				0.012‡
Currently employed	69	16 (34.0)	53 (18.2)	
Unemployed	269	31 (66.0)	238 (81.8)	
Parity				0.516‡
≤2	153	19 (40.4)	134 (46.0)	
≥3	185	28 (59.6)	157 (54.0)	
Household monthly income (Philippine peso)				0.099‡
≤5,000.00	216	25 (53.2)	191(65.6)	
>5,000.00	122	22 (46.8)	100 (34.4)	
Friend or family diagnosed with cervical cancer				0.135‡
Yes	10	3 (6.4)	7 (2.4)	
No	328	44 (93.6)	284 (97.6)	
Time to reach health facility for screening				0.258‡
60–120 min	154	25 (53.2)	129 (44.4)	
>120 min	184	22 (46.8)	162 (55.6)	
Contraceptive use				0.056‡
Yes	272	33 (70.2)	239 (82.1)	
No	66	14 (29.8)	52 (17.9)	
Knowledge score, median (range)	338	7 (1–11)	7 (0–12)	0.516§
Perceived susceptibility score, median (range)	338	22 (12–30)	23 (9–30)	0.441§
Perceived severity score, median (range)	338	35 (23–45)	35 (17–45)	0.712§

†, Cochran–Armitage test; ‡, Pearson chi-square test; §, Mann–Whitney U test.

**Table 4:** Factors Associated with Cervical Cancer Screening Utilization

Variable	Adjusted odds ratio (95% CI)	p-value
Age, years		
20–29	1	
30–39	3.76 (1.53–9.22)	0.004
≥40	6.58 (2.47–17.60)	<0.001
Education		
No formal education or primary	1	
Secondary and higher	4.20 (2.10–8.44)	<0.001

CI, confidence interval.

## Discussion

The rate of screening utilization was 13.9% among study participants, even though most participants (93.8%) had heard of screening. Many participants (71.3%) had heard of screening from a health facility. In the past, non-government organizations partnering with the local government have done cervical screening but it was not sustained due to financial constraints. The screening rate in this study was higher than that reported in a previous study among Filipino women aged 25–64 years indicating 9.3% coverage for those who received a Pap smear in the 3 years preceding being interviewed (WHO, 2002). However, the screening rate in the current study was lower than the other study findings which were 18.5% and 37% (Ngelangel et al., 1993; University of the Philippines- Department of Health Cervical Cancer Screening Study Group, 2001). The rates in these studies vary due to differences in study population, age range, screening period, and means of data collection.

In the current study, knowledge regarding HPV and cervical cancer was limited among participants. The mean knowledge score was 6.4 out of 15. Using the same questionnaire, previous studies conducted in the United States (US) obtained similar results. Ingledue et al., (2004) examined knowledge among female college students aged 18–30 years (mean score of 6.8). Lambert et al., (2015) reported a similar result among HIV-infected women aged 18–70 years (mean score of 6.02). Other research conducted in the US showed higher knowledge, with mean scores between 7.44 and 10.2 (Montgomery and Smith-Glasgow, 2012; Aleshire et al., 2013). A study among college students in health related courses in Philippines noted poor knowledge regarding transmission of HPV and diseases caused by HPV (Kiprono et al., 2012). The low level of knowledge about HPV and cervical cancer among our study participants needs to be addressed through educational intervention in health facilities and communities. 

In this study, the participants had higher scores for perceived susceptibility and severity than those in a previous study (Ingledue et al., 2004). Our participants believed HPV and cervical cancer to be a serious disease and that they were at risk of getting the disease. However, they took no action despite the perceived susceptibility of the disease. Ingledue et al., (2004) found mean scores of susceptibility and severity 17.4 and 24.8, respectively, whereas we obtained scores of 21.9 and 34.2, respectively, in the current study.

Logistic regression analysis revealed that age and education level were associated with women’s screening utilizations. Women who were older were more likely to have screening than younger women. Older women have more opportunities to obtain information or a recommendation for screening through utilization of health services such as antenatal care, child health care, and other health consultations. This result is consistent with previous studies that show age as one of the significant factors for women to go for screening (Boonpongmanee and Jittanoon, 2007). However, a study by Dunn and Tan (2010) showed that the relationship between age and screening utilization appears to increase first up to 40 years of age and then decrease. We also found that women with higher education levels were more likely to have had previous screening. Understandably, education is an important factor influencing health behavior. This finding was consistent with other research indicating a positive relationship between education status and screening utilization (Boonpongmanee and Jittanoon, 2007; Dunn and Tan, 2010). 

There are many studies indicating a positive correlation between knowledge, perceived susceptibility and severity, and having cervical cancer screening (Khani Jeihooni et al., 2015; Parsa et al., 2017). Other studies found that women have adequate knowledge of perceived susceptibility or severity but still do not utilize screening service (Srisuwan et al., 2015; Esike et al., 2018). The current study did not indicate a positive correlation between these factors. One possible reason for this might be that knowledge and perceptions of the disease were not a driving force for engaging in preventive behavior among our participants. Other than knowledge and perceptions, the perceived benefits and barriers to a recommended health action and self-efficacy also influence preventive behavior (Glanz et al., 2008). Moreover, demographic, psychosocial, and structural variables such as personal experience of disease may affect an individual’s perception and thus indirectly influence health-related behavior (Glanz et al., 2008; Limbu et al., 2018). Further research is needed to explore these associations. 

In our study, reasons for not having screening were mainly due to lack of money, having no signs or symptoms, fear of pain or discomfort and/or embarrassment during the procedure, having not heard of or not understanding the meaning of the screening test, and not being promoted. Similar findings have been reported in other Asian countries (Boonpongmanee and Jittanoon, 2007; Dunn and Tan, 2010; Jia et al., 2013; Darj, et al., 2019). The most frequent barrier to screening stated by participants was lack of money. This might be attributed to limited access to screening services or not being aware of screening cost. As in other developing countries, the availability of screening services is insufficient in rural areas due to resource constraints such as lack of trained personnel and equipped laboratories and being dependent on the direction of local government health priorities and financial support. Although screening service are provided free or at minimum cost, transport cost and time might be a burden on women. It is crucial to address improvement in access to screening services in the rural setting. One possible method to increase access to screening is to integrate screening services as part of the routine health care services (Bradley et al., 2005). This would minimize the burden of travel costs and time for these rural women. Although there might be health centers where screening services are already integrated in the existing health services, more effort is needed to increase utilization. Health care providers are expected to fully use all opportunities to increase screening utilization through about the routine services as well as to educate women, including the cost of screening. This could be an effective strategy in settings where there are no organized screening programs.

Another frequent statement was that participants did not undergo screening because they had no symptoms or signs such that they felt it was unnecessary to be screened or no benefit. These women may not know that HPV infection is usually asymptomatic (Chelimo et al., 2013) and cervical cancer is detectable by screening before symptoms develop. It is also noteworthy that fear of pain or discomfort of procedure or embarrassment related to screening were stated by many participants who had never undergone screening, which is similar to findings of community-based studies in other developing countries (Claeys et al., 2002; Twinomujuni et al., 2015). These results underscore the importance of designing educational or awareness interventions to address the perceived barriers. Educating women about the nature and progression of cervical cancer, benefits of screening, screening cost, and screening procedure, in addition to basic knowledge about the disease, such as its cause and symptoms, is essential to increase utilization of screening. 

The main reason for attending screening in our study was a request or recommendation of a health professional. Similar findings were reported in Malaysia. A qualitative study conducted by Wong et al., (2009) found that most respondents had never been recommended cervical cancer screening during their visits to health care providers. However, many said they would agree to have screening if their health care provider recommended it. Other studies also reported their recommendation was the most important driver to undergo screening (Okunowo and Smith-Okonu, 2020). As the Philippines relies on opportunistic screening program, the role of health care provider is critical in utilization of the service. Thus, health care providers need to be taught about the importance of their role in educating and motivating women to be screened. 

This study has several limitations. Screening utilization was investigated using self-reports, which could lead to recall bias. Participants may not be able to differentiate cervical screening from any pelvic examination or vaginal swab tests for infection (Gichangi et al., 2003), which would lead to overestimation of the screening rate. Despite these limitations, our study is one of the few studies on HPV, cervical cancer and screening conducted in the Philippines. The study results may provide a basis for improving current cervical cancer prevention programs.

In conclusion, the present study revealed that cervical cancer screening utilization among rural women in the Philippines was low. Although most women have heard of cervical cancer screening, their knowledge of HPV and cervical cancer is limited. Women’s health education needs to be strengthened to increase knowledge about the nature and progression and the benefits of cervical cancer screening even in asymptomatic women, screening cost, and the awareness of the screening procedure. Health care providers play an important role in educating and motivating women to increase screening utilization. Additional efforts are needed to increase the access to screening services in rural and remote areas of the Philippines such as Ticao island, Masbate. 
